# Shifts in Clinical Practice–Changing Acute Ischemic Stroke Research Over the Past Decade

**DOI:** 10.1161/SVIN.126.002479

**Published:** 2026-08-07

**Authors:** Mahnoor Khalid, Connor Nguyen, Jack Li, Aditya Bala, Tudor G. Jovin, Ashutosh P. Jadhav, Ngoc Mai Le, Javier Gomez Farias, Freya Kanakhara, Eunyoung A. Lee, David S. Liebeskind, Joseph N. Samaha, Hussain Azeem, Bruna Kfoury, Aman Yarlagadda, Sunil A. Sheth

**Affiliations:** Department of Neurology, McGovern Medical School at UTHealth, Houston, TX (M.K., A.B., N.M.L., J.G.F., F.K., E.A.L., J.N.S., H.A., B.K., S.A.S.).; Department of Neuroscience, Rice University 3990 , Houston, TX (C.N., J.L., A.Y.).; Department of Neurology, Cooper Medical School of Rowan University, Camden, NJ (T.G.J.).; Department of Neurology and Neurosurgery, University of Pittsburgh School of Medicine, PA (A.P.J.).; Departments of Neurology, Neurological Surgery, and Radiology, University of Southern California 5116 , Los Angeles (D.S.L.).

**Keywords:** American Heart Association, biomedical research, health expenditures, ischemic stroke, thrombolytic therapy

## Abstract

**BACKGROUND:**

The past decade has witnessed rapid growth of clinical trial programs in Europe and Asia, with randomized clinical trials (RCTs) publications from these regions outpacing those of the United States. However, limited data exist quantifying their relative influence on practice-defining results. Here, we evaluate these shifts by analyzing the geographic origin, funding source, and clinical impact of practice-changing RCTs.

**METHODS:**

From the 2018 and 2026 American Heart Association/American Stroke Association Acute Ischemic Stroke guidelines, we identified RCTs supporting new recommendations and extracted geographic origin (China/Europe/United States/Other), funding source (government/academic/nonprofit versus industry (private/mixed); National Institutes of Health versus non-National Institutes of Health), and research topic (endovascular therapy, thrombolysis, imaging, poststroke care, and prehospital and systems of care). Analyses used unweighted, reference-density-weighted, and clinical-impact-weighted strategies. Temporal trends were assessed using the χ^2^/Fisher exact tests, with the Rao-Scott adjusted χ^2^ tests accounting for weighting.

**RESULTS:**

We identified 21 new recommendations (47 RCTs) in 2018 and 45 (89 RCTs) in 2026. In 2018, Europe led (51.1%), followed by the United States (31.9%), while China and other regions contributed minimally. By 2026, Europe remained first (36%), China rose to second (29.2%), and the United States declined to the smallest share (14.6%), across all weighted analyses (*P*<0.01). National Institutes of Health–funded trials declined significantly from 21.3% (unweighted), 27.4% (reference-density-weighted), and 27.3% (clinical-impact-weighted) in 2018 to 4.5%, 4.8%, and 3.5% respectively in 2026 (*P*<0.01 across all weighted strategies).

**CONCLUSIONS:**

In this analysis, we identify a shift in practice-guiding research away from United States–based clinical trials and increasingly towards Europe and China. United States–based RCTs fell from the second most cited to the least cited sources of recommendations. National Institutes of Health–funded RCTs fell from nearly one-quarter of guideline-cited RCTs in 2018 to <5% in 2026, highlighting increasing reliance on non-US studies to inform US clinical care.

## Graphical abstract

**Figure:**
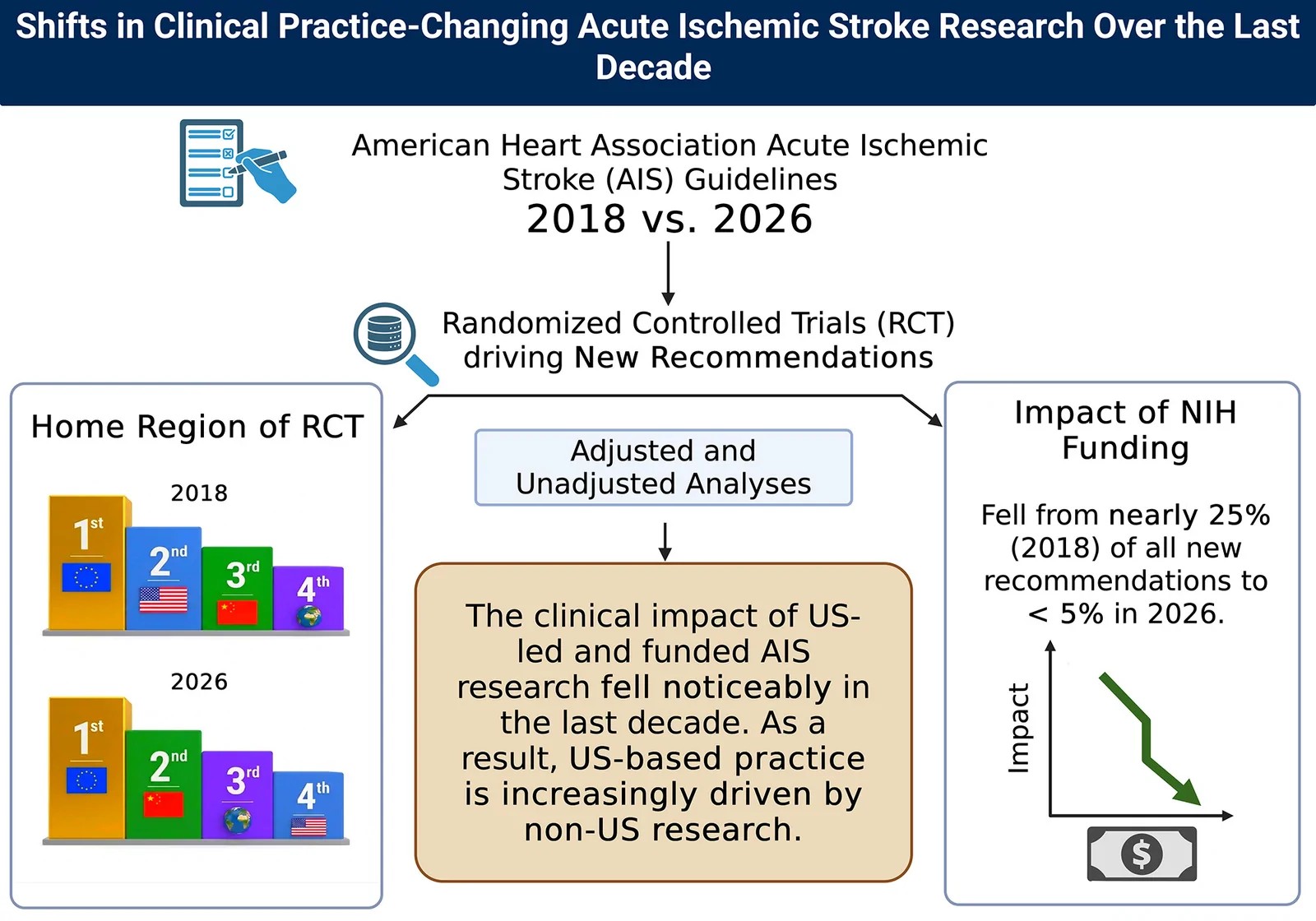

**CLINICAL PERSPECTIVE**

**What Is New?**

- Compared with the 2018 American Heart Association/American Stroke Association Acute Ischemic Stroke guidelines, the 2026 evidence base shifted away from United States–based and National Institutes of Health–funded randomized clinical trials and toward Europe and China, with the change consistent across weighting approaches, demonstrating that these changes reflected a transition in the total number of studies and their total impact.

**What Are the Clinical Implications?**

- Acute ischemic stroke practice in the United States is increasingly shaped by non-US trial data, highlighting the need for US clinicians and researchers to incorporate high-impact, globally competitive trial results for local implementation.

The global clinical research landscape has changed markedly over the past decade. Trial outputs from Europe and Asia, particularly China, have grown rapidly, with China surpassing the United States in both research expenditure and volume of scientific publications.^1^ These shifts have resulted from increased international research collaboration, investments in trial infrastructure, and policy efforts to strengthen research integrity.^2–4^ As a result, international trials are now being initiated and published faster than those based in the United States.^5^

Although the United States remains a leading contributor in biomedical research overall, its current role in acute ischemic stroke (AIS) research, as well as how the role has changed over time, remains incompletely characterized. Furthermore, because the raw number of trials being published is not necessarily an accurate indicator of the realized clinical impact, more nuanced approaches are necessary. For example, a trial evaluating a clinical question with study design and results comparable to 4 or 5 preceding trials may add only incremental value. As such, additional metrics are needed to weigh the overall influence of these studies when ascertaining their value.

To study the global landscape of clinical practice defining acute AIS research, we analyze data from widely accepted standard-defining documents, the American Heart Association (AHA)/American Stroke Association (ASA) AIS guidelines.^6^ Although previous studies have analyzed global trends in clinical trial registration and geographic distribution of stroke trials within the United States, no analysis has addressed the issue of overall clinical practice impact, particularly using multiple complementary weighting strategies.^7–9^ By analyzing the geographic origin, funding source, and clinical impact of randomized clinical trials (RCTs) that generated new recommendations in these guidelines, we identify evolving international trends in sources of evidence.

## Methods

### Data Availability

All data supporting the findings of this study are available within the article and its online Supplemental Material. The Supplemental Material includes the full set of guideline recommendations analyzed, the corresponding cited RCTs, and all assigned clinical impact scores. A detailed data extraction data set containing complete extraction variables, including geographic origin, funding source, and study characteristics, was compiled as part of this study and is available from the corresponding author upon reasonable request.

#### Study Design and Guideline Selection

We conducted a retrospective comparative bibliometric analysis of the 2018 and 2026 editions of the AHA/ASA guidelines for the early management of patients with AIS. We evaluated temporal shifts in RCT-derived evidence supporting the new recommendations in each guideline edition. To ensure a balanced comparison between the 2 guidelines and to capture significant shifts in the global research landscape, the focused 2019 update to the 2018 guideline was excluded from analysis. Only the complete 2018 and 2026 guideline documents were included.

#### Reference Identification and Eligibility

We identified new recommendations from the 2018 and 2026 AHA/ASA AIS guidelines that were labeled as new or revised by the writing committees. Recommendations that were previously present but only reworded for clarity or carried over without substantive changes in clinical practice were not included. For each new recommendation, all cited references were reviewed in full, and eligibility was limited to RCTs. This restriction was applied because RCTs provide the highest level of clinical evidence and greatly impact the generation, strength, and wording of guideline recommendations. Observational studies, cohort studies, registry analyses, case-control studies, meta-analyses, systematic reviews, expert opinion statements, and other nonrandomized designs were excluded. References not directly supporting a newly designated recommendation were also excluded.

#### Data Extraction and Classification

For each included RCT, geographic origin was categorized as China, Europe, the United States, or other (countries outside Europe, China, and the United States: Australia, New Zealand, Japan, South Korea, Canada, and Chile). For multinational trials, geographic classification was based on the location of the coordinating center or the first author’s primary institutional affiliation. This approach was intentional and was used to maintain consistency across analyses and ensure a standardized framework for geographic categorization throughout the study. The funding source was determined from the acknowledgment and disclosure sections of the original publication, categorizing it as government/academic/nonprofit or industry (private/mixed) funding. The funding source was further stratified into National Institutes of Health (NIH) versus non-NIH sources. Each RCT was also classified into a primary research area, defined as endovascular therapy (EVT), thrombolysis, imaging, prehospital and systems of care, or poststroke care. These categories were determined by the corresponding category definitions within the guideline publication. Each eligible RCT was also categorized by the Class of Recommendation (I, IIA, IIB, and III) assigned to the recommendation within which it was cited.

#### Weighting Strategies

To differentiate between the raw number of RCT references and their relative influence on guideline development, we applied 3 analytic weighting strategies. First, we performed an unweighted analysis where each eligible RCT publication contributed equally, regardless of the total number of references cited in the recommendation it supported. Next, we conducted a reference-density-weighted analysis, in which each trial was weighted as 1 divided by the total number of RCTs cited for that recommendation to account for citation density. As an example, in this analysis, if 4 trials were cited for a single recommendation, each trial was assigned a weight of 0.25. Finally, we performed a clinical-impact-weighted analysis, in which each new recommendation was assigned a clinical impact score from 1 (minimal expected impact on clinical practice) to 5 (major expected impact on practice). Clinical impact scores represented a subjective evaluation and were independently assigned by 2 board-certified vascular neurologists with over 15 years of experience in stroke care and involvement in guideline development. Both evaluators were blinded to each other’s assessments during the scoring process to minimize bias and ensure independent evaluation of each recommendation’s clinical impact. The average of the 2 reviewers’ scores was calculated and used as the final clinical impact score for each recommendation. To generate a clinical-impact-weighted measure for each RCT, this final clinical impact score was multiplied by the RCT’s reference-density-weight. Greater scores were assigned to recommendations with a higher class of recommendation, level of evidence, and their perceived influence on practice-changing recommendations. Recommendations that lead to paradigm shifts in acute therapy (ie, expanded thrombolysis window and expanded indications for EVT), which may result in system-wide changes in care, received higher scores relative to recommendations advising against interventions that were already not routinely performed before publication (eg, routine use of hypothermia in stroke or routine use of sonothrombolysis as an adjuvant to thrombolysis). The full list of recommendations, their cited RCTs, and their assigned clinical impact scores can be found in Table S1.

#### Statistical Analysis

We evaluated temporal differences between the 2018 and 2026 guidelines across geographic distribution, funding source composition, NIH versus non-NIH funding, and research area focus. All comparisons were conducted separately for unweighted, reference-density-weighted, and clinical-impact-weighted analyses. For unweighted analyses, categorical variables were compared using the Pearson χ^2^ test or the Fisher exact test as appropriate. For weighted analyses (reference-density-weighted and clinical-impact-weighted), the Rao-Scott adjusted χ^2^ test (second-order correction) was applied to account for noninteger cell values and the weighting structure, ensuring that *P* values appropriately reflect the design effect introduced by weighting. All statistical analyses were performed using Stata (version 19.2; StataCorp LLC, College Station, TX), with a 2-sided *P* value of <0.05 considered statistically significant.

## Results

**Figure 1.**
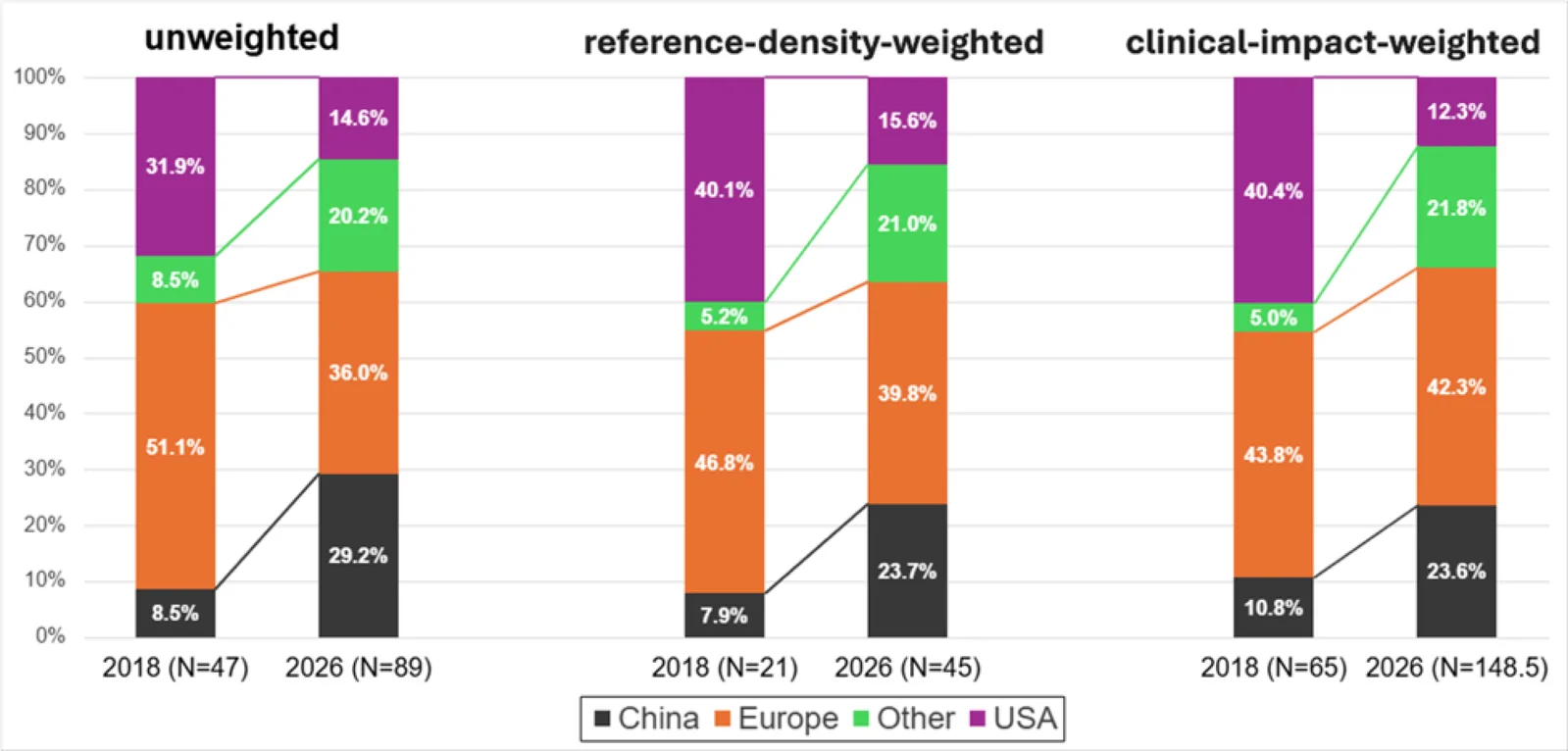
**Geographic distribution of randomized controlled trials (RCTs) cited in the 2018 and 2026 American Heart Association/American Stroke Association Acute Ischemic Stroke guidelines.** Stacked bar graphs show the distribution of RCTs cited in the 2018 and 2026 guidelines across 3 analytic approaches: unweighted analysis, reference-density-weighted analysis, and clinical-impact-weighted analysis. Within each analytic approach, bars represent the percentage contribution of trials from China, Europe, the United States, and other regions. Other includes trials conducted in countries outside the United States, China, and Europe (Australia, New Zealand, Japan, South Korea, Canada, and Chile). Differences between guideline eras were statistically significant (unweighted analysis: *P*=0.002; reference-density-weighted analysis: *P*=0.003; and clinical-impact-weighted analysis: *P*=0.002). Percentages were calculated using the number of RCT references in each category (numerator) divided by the total number of RCT references supporting new recommendations for that year and analytic approach (denominator): unweighted (2018: N=47; 2026: N=89), reference-density-weighted (2018: N=21; 2026: N=45), and clinical-impact-weighted (2018: N=65; 2026: N=148.5).

We identified 21 new recommendations in the 2018 guidelines and 45 in the 2026 update. These recommendations were supported by 47 RCTs in 2018 (class I: 6; class IIA: 10; class IIB: 22; and class III: 9) and 89 RCTs in 2026 (class I: 15; class IIA: 24; class IIB: 24; and class III: 26; Table S2). The geographic distribution of supporting RCTs shifted significantly between the 2 guideline eras across all weighting schemes (*P*<0.01; Figure 1). In 2018, Europe was the largest overall contributor to the RCT evidence base (51.1%), followed by the United States (31.9%), with China and other regions each contributing 8.5%. By 2026, Europe remained the leading contributor (36%); however, its proportional share decreased relative to 2018 (−15.1%) with similar directional decreases across weighted analyses. In contrast, China showed the largest positive shifts in contribution, rising from 8.5% in 2018 to 29.2% in 2026 (20.7%), becoming the second-largest contributor overall. Other regions also showed an increase in contribution from 8.5% to 20.2% (11.7%). The United States was the only group with a decline in both the absolute number of RCTs and overall proportional contribution (31.9%–14.6%; −17.3%), becoming the lowest contributor across all weighting schemes.

**Figure 2.**
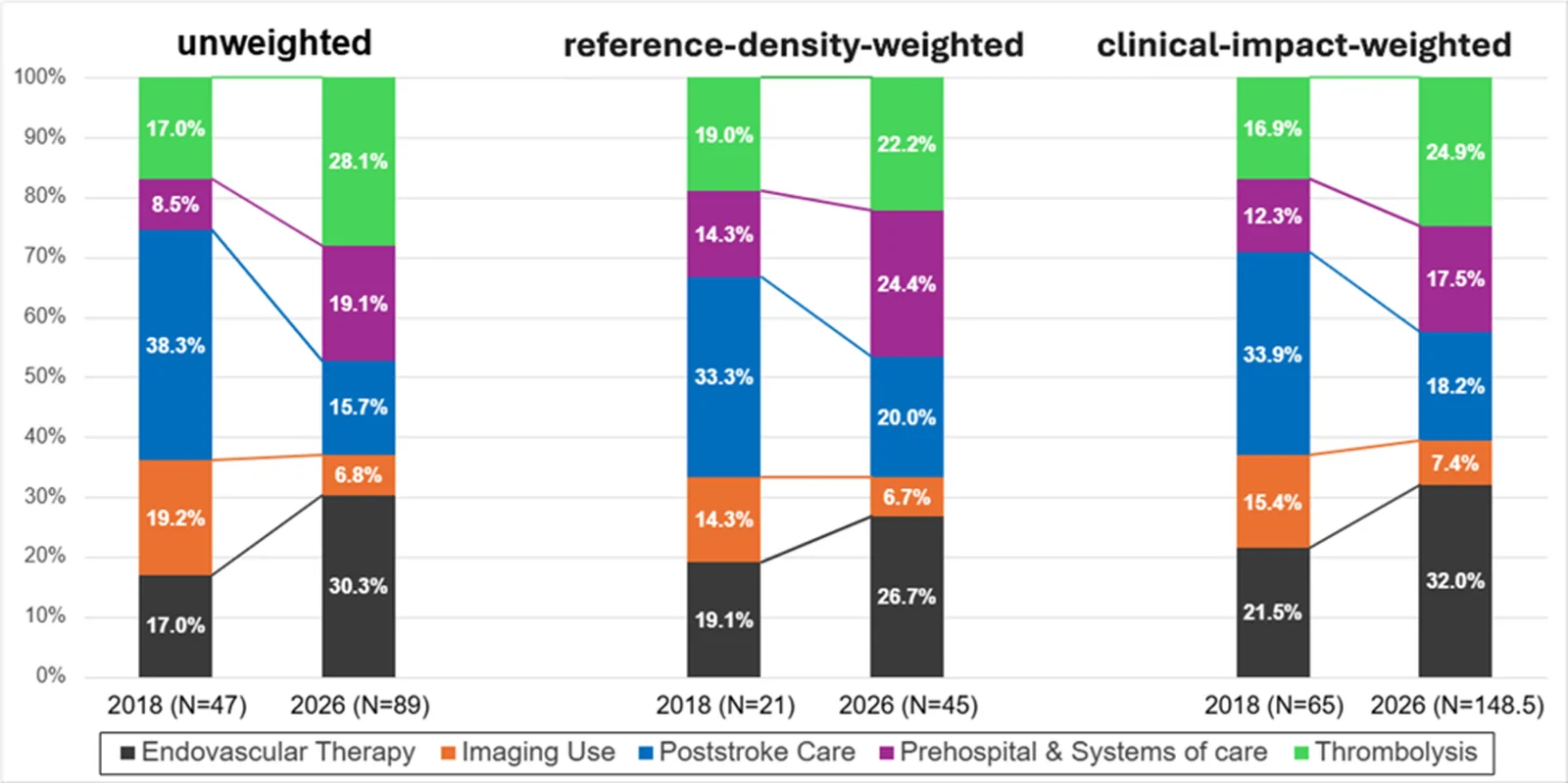
**Distribution of randomized controlled trials (RCTs) by research area cited in the 2018 and 2026 American Heart Association/American Stroke Association Acute Ischemic Stroke guidelines.** Stacked bar graphs show the distribution of RCTs cited in the 2018 and 2026 guidelines across 3 analytic approaches: unweighted analysis, reference-density-weighted analysis, and clinical-impact-weighted analysis. Within each analytic approach, bars represent the percentage of trials in each research area: endovascular therapy, thrombolysis, imaging, poststroke care, and prehospital and systems of care. Differences between guideline eras were significant for the unweighted analysis but not for the weighted analyses (unweighted: *P*=0.002; reference-density-weighted: *P*=0.326; and clinical-impact-weighted: *P*=0.258). Percentages were calculated using the number of RCT references in each category (numerator) divided by the total number of RCT references supporting new recommendations for that year and analytic approach (denominator): unweighted (2018: N=47; 2026: N=89), reference-density-weighted (2018: N=21; 2026: N=45), and clinical-impact-weighted (2018: N=65; 2026: N=148.5).

Research focus shifted significantly over time in unweighted analyses (*P*<0.01), while weighted distributions were not significant (*P*>0.05; Figure 2). In 2018, poststroke care was the most common research area (38.3%), followed by imaging (19.2%), EVT and thrombolysis (17% each), and prehospital and systems of care (8.5%). By 2026, EVT (30.3%) and thrombolysis (28.1%) became the top 2 areas, followed by prehospital and systems of care (19.1%), with poststroke care and imaging dropping to the lowest ranks (15.7% and 6.8%, respectively). Regional leadership within these research areas also changed markedly (Table 1). The United States decreased proportionally across all research areas, with the most prominent decline in imaging, dropping from the leading contributor (8.5%) in 2018% to 0% in 2026. China increased across almost all research areas except poststroke care, emerging as the top contributor in thrombolysis (0%–11.2%) and prehospital and systems of care (0%–6.7%), while Europe retained EVT leadership (8.5%–12.4%; 3.9%).

**Table 1.**
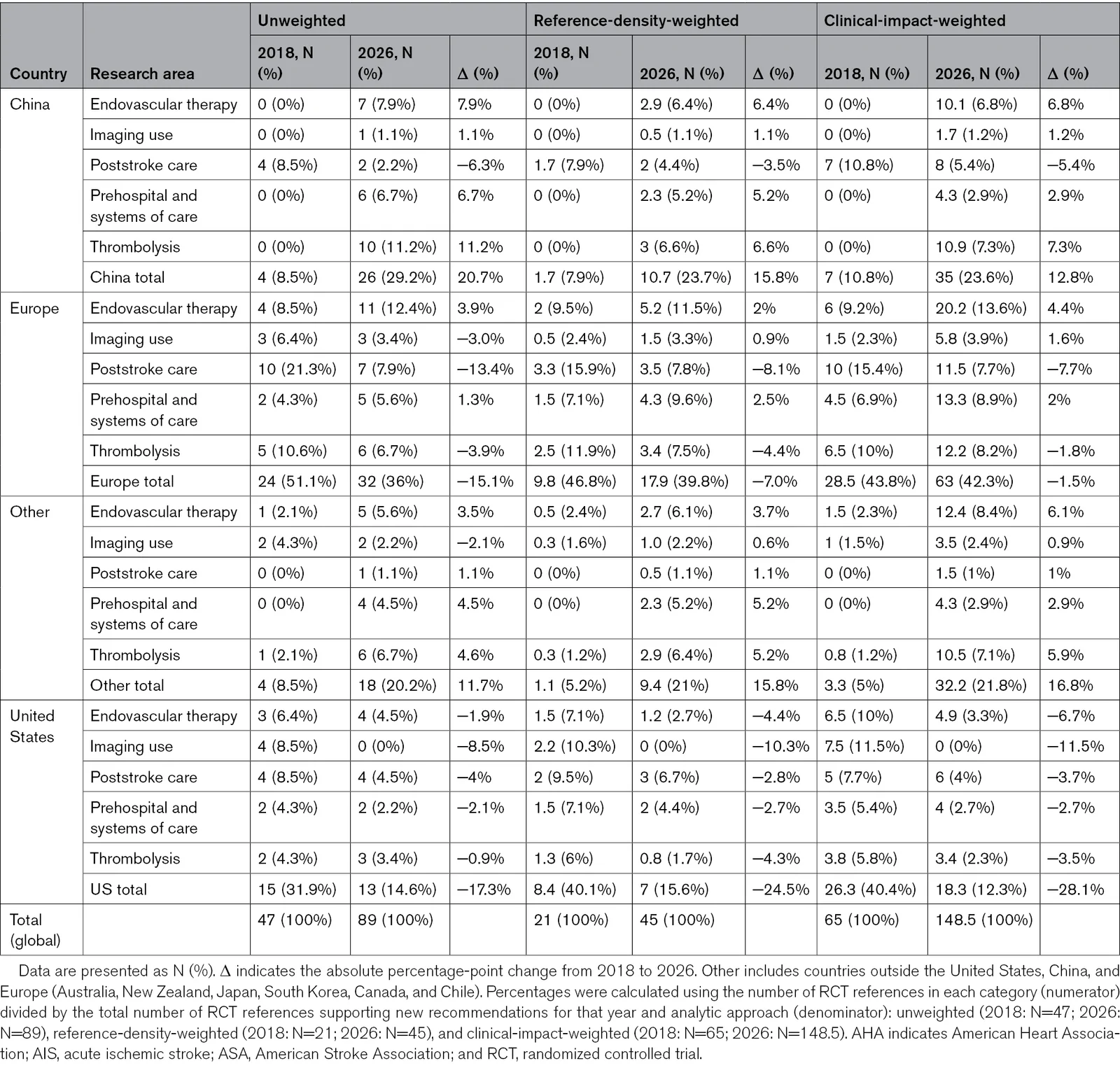
Geographic Distribution of RCTs Supporting New Recommendations in the 2018 and 2026 AHA/ASA AIS Guidelines, Stratified by Research Area

**Figure 3.**
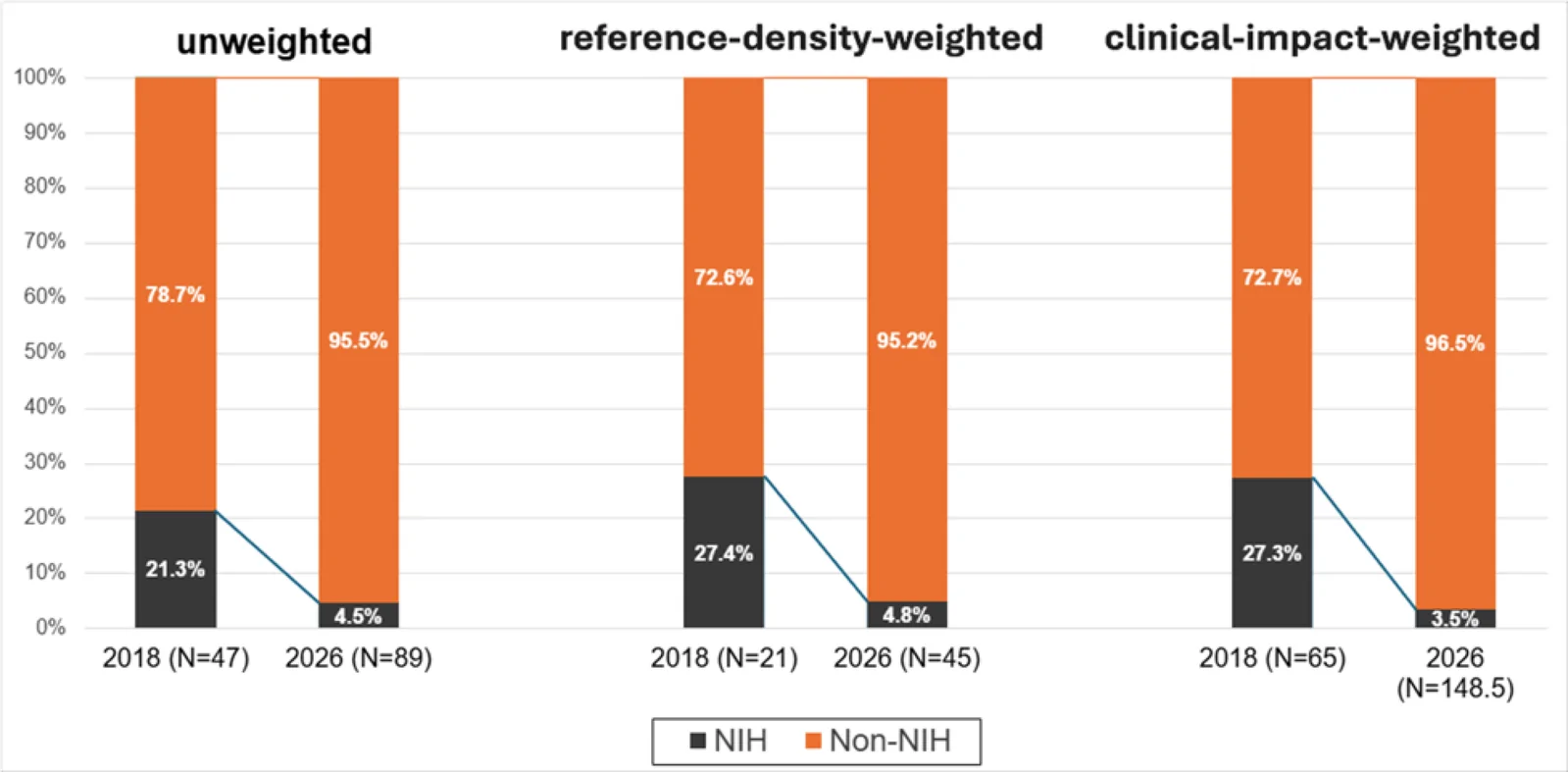
**Distribution of randomized controlled trial (RCT) funding by National Institutes of Health (NIH) vs non-NIH sources cited in the 2018 and 2026 American Heart Association/American Stroke Association Acute Ischemic Stroke guidelines.** Stacked bar graphs show the proportion of RCTs funded by NIH vs non-NIH sources globally for the 2018 and 2026 guidelines across 3 analytic approaches: unweighted analysis, reference-density-weighted analysis, and clinical-impact-weighted analysis. Overall, NIH-funding distributions differed significantly between guideline eras (unweighted: *P*=0.002; reference-density-weighted: *P*=0.002; and clinical-impact-weighted: *P*<0.001). Percentages were calculated using the number of RCT references in each category (numerator) divided by the total number of RCT references supporting new recommendations for that year and analytic approach (denominator): unweighted (2018: N=47; 2026: N=89), reference-density-weighted (2018: N=21; 2026: N=45), and clinical-impact-weighted (2018: N=65; 2026: N=148.5).

Funding patterns changed more subtly. Overall, government/academic/nonprofit funding remained the dominant source in both years (74.5% in 2018 versus 68.5% in 2026; −6.0%), with a corresponding increase in industry or mixed private funding (25.5%–31.5%; 6.0%), changes that were not statistically significant (*P*>0.05 across all weighting schemes; Figure S1). When broken down by research areas, government funding grew markedly in prehospital and systems of care (10.6%) and EVT (8.4%) but declined sharply in poststroke care (−23.9%), while industry showed gains in thrombolysis (6.9%) and remained absent from prehospital and systems of care (0% in both guideline years; Table 2). The overall proportion of NIH-funded RCTs was substantial in 2018 guidelines (21.3% unweighted, 27.4% reference-density-weighted, and 27.3% clinical-impact-weighted) but fell considerably and significantly by 2026 (4.5%, 4.8%, and 3.5%, respectively; *P*<0.01 across all comparisons; Figure 3).

**Table 2.**
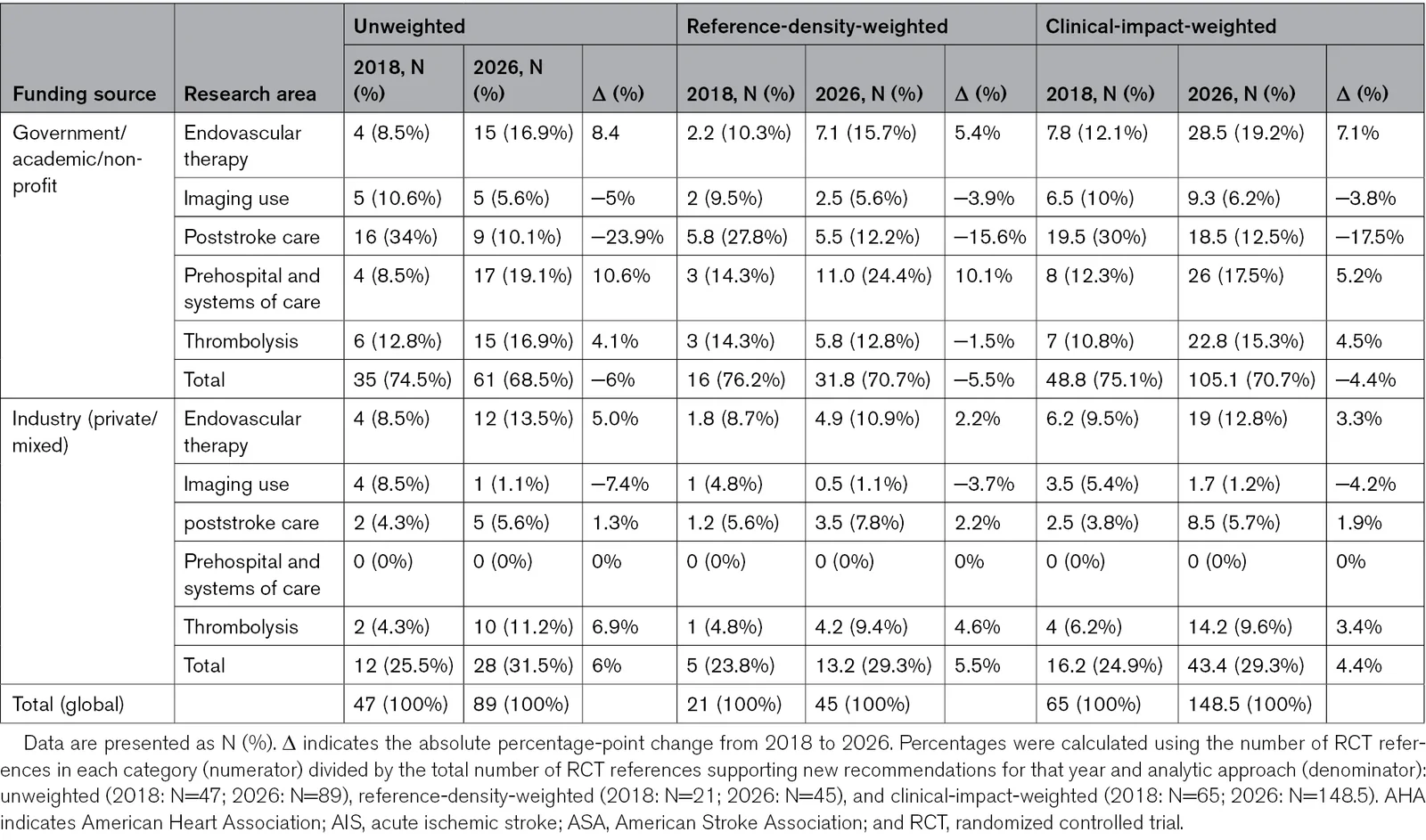
Funding Source of RCTs Supporting New Recommendations in the 2018 and 2026 AHA/ASA AIS Guidelines, Stratified by Research Area

## Discussion

In this analysis of global trends in practice-changing AIS research using the AHA/ASA AIS guidelines, we observed a clear shift in geographic distribution and funding patterns. Europe remained the largest source of RCT citations. On the other hand, whereas United States–based RCTs had represented the second highest number of citations for new recommendations in the 2018 guidelines, they contributed the least of the 4 regions in 2026. The United States was the only region to show a significant decrease in contribution across all 3 weighting approaches. In addition, whereas NIH-funded RCTs played an outsized role in new recommendations in 2018 (nearly one-fourth of all the new recommendations), their impact fell to <5% in 2026. Conversely, Chinese research became the second most cited source of evidence (nearly one-third of all references), with growth in nearly all the AIS research topics.

For many years, trials from China and other rapidly expanding research regions had been viewed with caution. Several studies have raised concerns about data accuracy, including documented higher retraction rates, incomplete trial registration, selective reporting, language and indexing biases, and systemic pressures to produce positive results.^10–14^ However, similar issues have been reported in other international data sets.^15,16^ In addition, differences in ethnic diversity, stroke cause (ie, greater rates of hemorrhagic stroke and intracranial atherosclerosis), and healthcare systems have led to questions around generalizability to United States–based populations.^17–20^ These issues had previously influenced how researchers evaluate studies and uphold standards of research integrity and external validity.^21^ However, the current analysis reveals that several of the most high-impact 2026 recommendations now rest primarily on international RCTs. A prominent example is basilar artery occlusion, where the class I recommendation for EVT within 24 hours is built soley on Chinese multicenter trials, including ATTENTION (Endovascular Treatment for Acute Basilar Artery Occlusion: A Multicenter Randomized Clinical Trial) and BAOCHE (Randomized Trial of Revascularization With Solitaire Stentriever vs Best Medical Therapy in the Treatment of Acute Ischemic Stroke due to Basilar Artery Occlusion Presenting Within 6-24 Hours of Symptom Onset).^22,23^ These trials were conducted in large, specialized stroke centers with high patient volumes and centralized systems that facilitate rapid enrollment and execution. As such, whereas concerns regarding the validity and generalizability of Chinese RCTs may have previously led to hesitancy in their use for practice guideline generation, this reluctance may no longer be sustainable given the difficulty of generating comparable United States–based evidence for such vital interventions. It may be more productive to consider these trials within the same methodological and ethical framework applied to trials in other regions. Structural and demographic factors, such as larger at-risk populations and efficient care networks, may also facilitate large-scale, high-quality AIS research in certain settings.

The relative decrease in NIH-funded trials and their clinical impact within guideline-cited RCTs is particularly notable. In 2018, NIH-supported trials, such as NINDS rt-PA (National Institute of Neurological Disorders and Stroke rt-PA), INSTINCT (Clinical Trial to Increase t-PA Use in Stroke Treatment), and NIH-backed components of DEFUSE 3 (Endovascular Therapy Following Imaging Evaluation for Ischemic Stroke 3), accounted for roughly one‑quarter of guideline‑driving RCTs globally and two-thirds of United States-origin trials.^24–26^ By 2026, citations to NIH-funded trials dropped across all weighting strategies, despite the continued operation of StrokeNet, established in 2013 to facilitate multicenter stroke research. On NIH RePORTER (NIH Research Portfolio Online Reporting Tool), the total funding for StrokeNet has not been substantially reduced from the period of 2018 to 2025, averaging around $30 million per year throughout this time period.^27^

At the same time, structural and systemic factors may further limit the representation and clinical impact of United States–based stroke trials. Although the United States continues to register the largest number of clinical trials globally, including in AIS, growth in AIS trials has been more modest compared with other fields. AIS research remains unevenly distributed within the country, with several high-burden regions, such as Mississippi and Louisiana, underrepresented.^7,9^ The sharper decline in United States–based RCT impact under clinical-impact-weighting may reflect the fact that many US/NIH stroke trials have been duplicated in other settings or that United States–based studies targeted less impactful questions. These structural imbalances, combined with rising trial costs and recruitment challenges particularly in time-sensitive AIS populations, may further limit the efficiency and clinical impact of United States–based AIS trials. Previous studies have demonstrated that US government–funded trials are associated with longer overall trial timelines and delayed dissemination compared with industry-funded studies. These differences may reflect a combination of regulatory, administrative, and oversight-related factors, as well as differences in trial design characteristics. Federally funded trials also tend to enroll fewer participants and are more frequently conducted as single-site studies, which may influence trial conduct and reporting timelines.^28,29^ Differences in population size and healthcare system structure may also contribute to more rapid patient recruitment and trial completion in some international settings, where centralized systems and larger patient volumes facilitate large-scale enrollment.^30^ Furthermore, although nominal federal funding levels may seem relatively stable, rising healthcare costs, administrative requirements, and operational expenses may affect the effective purchasing power of research awards, potentially limiting the scale or number of trials that can be supported. These issues may continue to compound, given the substantially lower trial costs outside North America (49%–78% of US costs per site).^31–34^

This shift carries immediate implications for US practice. Clinicians now rely heavily on non-US trials for routine decisions about reperfusion therapies, patient selection, and systems of care. While US clinicians benefit from expanded high-quality global evidence, reduced domestic representation within guideline-defining research reflects structural and competitive factors. Reference selection in guidelines is inherently subjective, with authors often favoring studies from their own institutions, collaborators, or home countries.^35,36^ In our data set, both the 2018 and 2026 AIS guidelines were written by predominantly United States–based groups, conditions under which selection bias would most likely favor US- and NIH-funded trials.^37^ Yet, United States-origin RCTs lost ground across all weighted analyses, and the relative influence of NIH-funded AIS trials dropped even more steeply. Improving the translational efficiency of NIH‑ and StrokeNet-supported research will be critical to preserving US leadership in evidence generation, perhaps by prioritizing high-impact trials that are both feasible and positioned to meaningfully change care and compete globally.^38^

Importantly, these trends should not necessarily be interpreted solely as a decline in US research output. Rather, they reflect a shift in the relative contribution of different regions to guideline-cited RCTs within an increasingly competitive global research ecosystem. High-quality evidence is now being generated across multiple international settings, and this diversification has likely strengthened the overall evidence base informing AIS care. Some regions may currently be better positioned to conduct certain studies because of differences in patient volume, trial efficiency, healthcare system structure, and scalability, facilitating the completion of trials that may have been difficult to conduct elsewhere.

There are limitations to this study that should be acknowledged. First, we analyzed only guideline-cited RCTs although high-quality observational cohorts, registry data, and mechanistic studies also influence recommendations. We made this choice intentionally because non-RCTs derive lower classes of recommendation, and our objective was to evaluate practice-changing research. Next, we examined only 2 guideline time points, which provide a snapshot rather than a continuous trajectory across the intervening years. However, these 2 guidelines represent RCTs that span over a decade and likely capture longer-term trends. Third, we did not formally assess trial quality or risk of bias, relying instead on guideline inclusion as our quality filter. It is important to recognize that not all RCTs are practice-changing. Federally funded studies may more frequently address clinically important but higher-risk or confirmatory questions, which can yield neutral or negative results. Although such trials remain scientifically valuable, they may be less likely to be incorporated into guideline-defining recommendations or achieve high citation impact. However, because our inclusion criteria were applied uniformly across all regions and funding sources, any temporal changes in the proportion of neutral or negative trials would be expected to affect all geographic groups similarly. We also did not evaluate research integrity issues by country or sponsor. In addition, for multinational trials, geographic classification was based on the location of the coordinating center or the first author’s primary institutional affiliation. This approach may not reflect the international and collaborative nature of contemporary RCTs and may not capture contributions across regions, particularly in large multicenter studies. This approach was intentional and was used for consistency across analyses. Finally, our broad funding categories (NIH versus non-NIH; government/academic/nonprofit versus industry) simplify complex funding arrangements and may mask important differences in how public and private entities collaborate on individual trials. Nevertheless, RCTs in AHA guidelines are not random. They represent the studies that expert groups judge to be most relevant for defining the standard of care and often influence certification requirements, quality metrics, and reimbursement decisions.

In this bibliometric analysis of the AHA/ASA AIS guidelines, we identified a large contribution of clinical practice–changing United States–based research and NIH-funded RCTs in 2018, which fell considerably in 2026. We also identified a converse increase in RCTs from China. Europe remained the dominant region. These findings highlight the need to strengthen the US clinical trial ecosystem to ensure continued leadership and meaningful contribution within an increasingly collaborative and international research landscape.

## ARTICLE INFORMATION

### Sources of Funding

Dr Sheth has been supported in part by the National Institutes of Health grants R01NS121154 and R01NS138765.

### Disclosures

Drs Jovin and Liebeskind serve on the editorial board of *Stroke: Vascular and Interventional Neurology* (*S:VIN*). Editorial board members are not involved in the handling or final disposition of submissions. Dr Jadhav is the Editor-in-Chief for *S:VIN* and was not involved in the handling or final disposition of this article. Disclosures provided by Dr Jadhav, in compliance with American Heart Association’s annual Journal Editor Disclosure Questionnaire, are available at https://www.ahajournals.org/editor-coi-disclosures. Dr Sheth is an Associate Editor for *S:VIN* and was not involved in the handling or final disposition of this article. Disclosures provided by Dr Sheth, in compliance with American Heart Association’s annual Journal Editor Disclosure Questionnaire, are available at https://www.ahajournals.org/editor-coi-disclosures. The other authors report no conflicts.

### Supplemental Material

Tables S1–S2

Figure S1

## Supplementary Material


