# Supplementary material for: Shifts in Clinical Practice–Changing Acute Ischemic Stroke Research Over the Past Decade
Source: Stroke Vasc Interv Neurol. 2026 Aug 7;6(5):e002479. doi: 10.1161/SVIN.126.002479 (PMC13549562; doi:10.1161/SVIN.126.002479)
Supplement: Supplementary file 1 [file svi2-6-e002479-s001.docx]

**Supplemental Material**

**Shifts in Clinical Practice-Changing Acute Ischemic Stroke Research Over the Last Decade**

Mahnoor Khalid^1^, Connor Nguyen^2^, Jack Li^2^, Aditya Bala^2^, Tudor G. Jovin^3^, Ashutosh P. Jadhav^4^, Ngoc Mai Le^1^, Javier Gomez Farias^1^, Freya Kanakhara^1^, Eunyoung A. Lee^1^, David S. Liebeskind^5^, Joseph N. Samaha^1^, Hussain Azeem^1^, Bruna Kfoury^1^, Aman Yarlagadda^2^, Sunil A. Sheth^*1^

^1^Department of Neurology, McGovern Medical School at UTHealth, Houston, Texas, USA

^2^Department of Neuroscience, Rice University, Houston, Texas, USA

^3^Department of Neurology, Cooper Medical School of Rowan University, Camden, New Jersey, USA

^4^Department of Neurology and Neurosurgery, University of Pittsburgh School of Medicine, Pittsburgh, Pennsylvania, USA

^5^Departments of Neurology, Neurological Surgery and Radiology, University of Southern California, Los Angeles, CA, USA

**Corresponding Author:**
Sunil A. Sheth, MD
6431 Fannin St, MSB 7.220
Houston, TX 77030, USA
Email: [sunil.a.sheth@uth.tmc.edu](mailto:sunil.a.sheth@uth.tmc.edu)
Tel: 713-500-7066

**Short title:** Shifts in AIS Research

**Keywords:** Acute Ischemic Stroke, Endovascular Thrombectomy, Thrombolytic Therapy, NIH Funding

**Total Word Count: 3677**

**Supplemental Table S1. New Recommendations in the 2018 and 2026 AHA/ASA AIS Guidelines with Clinical Impact Scores and Supporting RCTs**

| **Guideline Year** | **Recommendations** | **Clinical Impact Score** | | **Cited RCTs** |
| --- | --- | --- | --- | --- |
|  |  | **Neurologist 1** | **Neurologist 2** |  |
| **2018** | EMS personnel should begin the initial management of stroke in the field. Implementation of a stroke protocol to be used by EMS personnel is strongly encouraged. | 3 | 3 | Berglund et al., 2014 |
|  | Multicomponent quality improvement initiatives, which include ED education and multidisciplinary teams with access to neurological expertise, are recommended to safely increase IV thrombolytic treatment. | 3 | 3 | Scott et al., 2013; Dirks et al., 2011 |
|  | Stroke outcome measures should include adjustments for baseline severity. | 2 | 2 | Scott et al., 2013 |
|  | Routine use of magnetic resonance imaging (MRI) to exclude cerebral microbleeds (CMBs) before administration of IV alteplase is not recommended. | 3 | 3 | NINDS rt-PA Stroke Study Group et al., 1995 |
|  | Multimodal CT and MRI, including perfusion imaging, should not delay administration of IV alteplase. | 3 | 3 | Huang et al., 2015; Parsons et al., 2012; Hacke et al., 2009; Davis et al., 2008; Furlan et al., 2006; Hacke et al., 2005 |
|  | In selected patients with AIS within 6 to 24 hours of last known normal who have LVO in the anterior circulation, obtaining CTP, DW-MRI, or MRI perfusion is recommended to aid in patient selection for mechanical thrombectomy, but only when imaging and other eligibility criteria from RCTs showing benefit are being strictly applied in selecting patients for mechanical thrombectomy. | 4 | 4 | Nogueira et al., 2017; Albers et al., 2018 |
|  | For otherwise eligible patients with mild stroke presenting in the 3- to 4.5-hour window, treatment with IV alteplase may be reasonable. Treatment risks should be weighed against possible benefits. | 4 | 4 | Hacke et al., 2008; Bluhmki et al., 2009 |
|  | In otherwise eligible patients who have had a previously demonstrated high burden of CMBs (>10) on MRI, treatment with IV alteplase may be associated with an increased risk of sICH, and the benefits of treatment are uncertain. Treatment may be reasonable if there is potential for substantial benefit. | 3 | 3 | NINDS rt-PA Stroke Study Group et al., 1995 |
|  | Tenecteplase administered as a 0.4-mg/kg single IV bolus has not been proven to be superior or noninferior to alteplase but might be considered as an alternative to alteplase in patients with minor neurological impairment and no major intracranial occlusion. | 3 | 3 | Huang et al., 2015; Parsons et al., 2012; Haley et al., 2010; Logallo et al., 2017 |
|  | The use of sonothrombolysis as adjuvant therapy with IV thrombolysis is not recommended. | 1 | 1 | Nacu et al., 2017 |
|  | In selected patients with AIS within 16 to 24 hours of last known normal who have LVO in the anterior circulation and meet other DAWN eligibility criteria, mechanical thrombectomy is reasonable. | 5 | 5 | Nogueira et al., 2017; Albers et al., 2018 |
|  | EVT of tandem occlusions (both extracranial and intracranial occlusions) at the time of thrombectomy may be reasonable. | 4 | 4 | Bracard et al., 2016 |
|  | It is reasonable to select an anesthetic technique during  endovascular therapy for AIS on the basis of individualized assessment of patient risk factors, technical performance of the procedure, and other clinical characteristics. Further randomized trial data are needed. | 2 | 2 | Berkhemer et al., 2016; Löwhagen Hendén et al., 2017; Schönenberger et al., 2016 |
|  | In patients who undergo mechanical thrombectomy with  successful reperfusion, it might be reasonable to maintain BP at a level <180/105 mm Hg. | 3 | 3 | Goyal et al., 2015; Jovin et al., 2017 |
| **2018** | In patients presenting with minor stroke, treatment for 21 days with dual antiplatelet therapy (aspirin and clopidogrel) begun within 24 hours can be beneficial for early secondary stroke prevention for a period of up to 90 days from symptom onset. | 5 | 5 | Wang et al., 2013; Johnston et al., 2013 |
|  | Ticagrelor is not recommended (over aspirin) in the acute treatment of patients with minor stroke. | 2 | 2 | Johnston et al., 2016 |
|  | In patients with BP ≥220/120 mm Hg who did not receive IV alteplase or EVT and have no comorbid conditions requiring acute antihypertensive treatment, the benefit of initiating or reinitiating treatment of hypertension within the first 48 to 72 hours is uncertain. It might be reasonable to lower BP by 15% during the first 24 hours after onset of stroke. | 3 | 3 | Horn et al., 2001; Schrader et al., 2003; Potter et al., 2009; Robinson et al., 2010; He et al., 2014; ENOS Trial Investigators et al., 2015 |
|  | Starting or restarting antihypertensive therapy during hospitalization in patients with BP >140/90 mm Hg who are neurologically stable is safe and is reasonable to improve long-term BP control unless contraindicated. | 3 | 3 | Robinson et al., 2010; He et al., 2014 |
|  | The benefit of induced hypothermia for treating patients with  ischemic stroke is not well established. Hypothermia should be offered only in the context of ongoing clinical trials. | 1 | 1 | Lyden et al., 2016; Geurts et al., 2017; Piironen et al., 2014; Hemmen et al., 2010 |
|  | For patients with dysphagia, it is reasonable to initially use nasogastric tubes for feeding in the early phase of stroke (starting within the first 7 days) and to place percutaneous gastrostomy tubes in patients with longer anticipated persistent inability to swallow safely (>2–3 weeks). | 3 | 3 | Dennis et al., 2006 |
|  | In patients >60 years of age with unilateral MCA infarctions who deteriorate neurologically within 48 hours despite medical therapy, decompressive craniectomy with dural expansion may be considered because it reduces mortality by close to 50%, with 11% of the surgical survivors achieving moderate disability (able to walk [mRS score 3]) and none achieving independence (mRS score ≤2) at 12 months. | 5 | 5 | Jüttler et al., 2014; Zhao et al., 2012 |
| **2026** | In patients with suspected AIS, the use of MSUs over conventional EMS where available is recommended for the transport and management of thrombolytic-eligible patients to ensure the fastest achievable onset-to-treatment time and improve functional outcomes. | 4 | 4 | Ebinger et al., 2014 |
|  | In patients with suspected acute stroke, MSUs must be equipped to diagnose and treat patients with IVT. | 2 | 2 | Walter et al., 2012 |
|  | In patients with suspected acute stroke, MSU care, including streamlined protocols and use of neurological expertise, either in-person or remote telemedicine consultation, is beneficial for emergent evaluation and treatment of patient symptoms without safety concerns. | 2 | 2 | Wu et al., 2017 |
|  | In endovascular thrombectomy-eligible patients, use of MSUs can be beneficial to identify and triage patients to the appropriate thrombectomy-capable facility with prehospital notification of receiving stroke teams. | 3 | 3 | Helwig et al., 2019 |
|  | For patients with suspected stroke in the prehospital setting, telemedicine in the ambulance should be considered, when feasible, to complement paramedic assessment to identify candidates for reperfusion interventions. | 2 | 2 | Scott et al., 2022 |
|  | In patients with suspected AIS who awaken from symptoms or have unknown time of onset >4.5 hours from last known well, but are otherwise eligible for thrombolysis, MRI DWI-FLAIR mismatch selection can be useful to determine eligibility for extended window IVT. | 4 | 3 | Thomalla et al., 2018; Koga et al., 2020 |
| **2026** | In patients with suspected AIS who awaken with symptoms or have unknown time of onset 4.5 to 24 hours from last known well, CTP or MR DWI-PWI (perfusion-weighted imaging) selection with automated postprocessing software analysis can be useful to determine eligibility for extended window IVT. | 4 | 3 | Ma et al., 2019; Xiong et al., 2024 |
|  | In patients with suspected AIS and LVO based on prehospital assessment with a validated stroke severity scale (eg, RACE >4) and eligible for EVT, direct triage to the angiography suite (DTAS) for flat-panel head CT versus conventional imaging workflow followed by catheter-based angiography may be considered to reduce time to intervention and improve functional outcomes. | 4 | 4 | Requena et al., 2021; Pfaff et al., 2020 |
|  | In patients with AIS within 6 hours from onset, NIHSS score 10 to 20, CT ASPECTS of ≥6, and anterior circulation LVO (M1 or carotid terminus) with planned EVT (with or without IVT) normobaric hyperoxia (NBO) before EVT may be reasonable to improve functional outcomes at 90 days. | 2 | 2 | Li et al., 2024; Cheng et al., 2022; Li et al., 2022; Li et al., 2025 |
|  | In patients with AIS, there is no benefit of 0-degree head  positioning compared with 30 degrees for 24 hours, to improve functional outcome. | 1 | 1 | Anderson et al., 2017; Olavarria et al., 2018 |
|  | In patients with AIS with probable large artery atherosclerosis etiology for whom no reperfusion intervention is available, there is no benefit of routine Trendelenburg positioning (-20 degrees) compared with 0- to-30 degree head positioning to improve functional outcome. | 1 | 1 | Chen et al., 2023 |
|  | In patients with mild to moderate severity AIS who have been treated with IVT, intensive SBP reduction (target of <140 mm  Hg compared with <180 mm Hg) is not recommended because it is not associated with an improvement in functional outcome. | 3 | 3 | Anderson et al., 2019 |
|  | In patients with AIS with LVO of the anterior circulation who have been successfully recanalized by endovascular therapy (mTICI 2b, 2c, or 3) and without other indication for blood pressure management target, intensive SBP reduction target of <140 mm Hg for the first 72 hours is harmful and not recommended. | 4 | 4 | Yang et al., 2022; Nam et al., 2023; Minhas et al., 2021 |
|  | In hospitalized patients with AIS with hyperglycemia, treatment with IV insulin to achieve blood glucose levels in the range of 80 to 130 mg/dL is not recommended to improve 3-month functional outcomes. | 2 | 2 | Johnston et al., 2019 |
|  | In eligible adult patients with AIS presenting mild non-disabling stroke deficits (eg, isolated sensory syndrome in many cases) within 4.5 hours of symptom onset or last known well, IVT is not recommended to improve functional outcomes. Instead, dual antiplatelet therapy (DAPT) should be considered* to reduce the risk of early recurrent stroke. | 5 | 5 | Xiong et al., 2025; Chen et al., 2023; Khatri et al., 2018; Coutts et al., 2024; Cui et al., 2024; Johnston et al., 2018; Wang et al., 2013 |
|  | In adult patients with AIS presenting within 4.5 hours of symptom onset or last known well and eligible for IVT, tenecteplase at a dose of 0.25 mg/kg body weight (max 25 mg) or alteplase at a dose of 0.9 mg/kg body weight is recommended to improve functional outcomes. | 5 | 5 | Muir et al., 2024; Menon et al., 2022; Wang et al., 2023; Parsons et al., 2024; Meng et al., 2024 |
|  | In adult patients with AIS presenting within 4.5 hours of symptom onset or last known well and eligible for IVT, tenecteplase at a dose of 0.4 mg/kg body weight is not recommended. | 4 | 4 | Kvistad et al., 2022; Logallo et al., 2017; Campbell et al., 2020 |
| **2026** | In patients with AIS who (a) have unknown time of onset and are within 4.5 hours from symptom recognition and (b) have an MRI-DWI lesion smaller than one-third of the MCA territory and no marked signal change on FLAIR, IVT administered within 4.5 hours of stroke symptom recognition can be beneficial to improve functional outcomes. | 5 | 4 | Thomalla et al., 2018 |
|  | In patients with AIS who (a) awake with stroke symptoms within 9 hours from the midpoint of sleep or are 4.5-9 hours from last known well and (b) have salvageable ischemic penumbra detected on automated perfusion imaging, IV thrombolysis may be reasonable to improve functional outcomes | 5 | 4 | Ma et al., 2019 |
|  | In patients with AIS due to LVO with salvageable ischemic penumbra, presenting within 4.5 to 24 hours from symptom onset or last known well, and who cannot receive EVT, treatment with IVT directed by individuals with expertise in thrombolytic stroke care may be beneficial to improve functional outcomes. | 4 | 4 | Ringleb et al., 2019; Albers et al., 2024 |
|  | In eligible patients with AIS presenting within 4.5 hours from last known normal and not undergoing EVT, IV reteplase, instead of alteplase, may be considered to increase the odds of excellent functional outcome at 90 days. | 3 | 3 | Li et al., 2024; Li et al., 2024 |
|  | In eligible patients with AIS within 4.5 hours from last known normal and not undergoing EVT, IV mutant prourokinase, instead of alteplase, may be considered due to lower odds of sICH and noninferiority for odds of excellent functional outcome at 90 days. | 3 | 3 | Song et al., 2023; Li et al., 2025 |
|  | In eligible patients with AIS within 4.5 hours from last known normal, IV mutant prourokinase in conjunction with low-dose alteplase is not recommended to improve functional outcomes. | 2 | 2 | van der Ende et al., 2023 |
|  | In adults with acute nonarteritic central retinal artery occlusion (CRAO) causing disabling visual loss, and who are otherwise eligible for IVT, the usefulness of treatment with IVT within 4.5 hours of time last known well is uncertain. | 2 | 2 | Chen et al., 2011 |
|  | In patients with AIS from anterior circulation proximal LVO of the ICA or M1, presenting within 6 hours from onset of symptoms, with NIHSS score ≥6, prestroke mRS score of 0 to 1, and ASPECTS 3 to 10, EVT is recommended to improve functional clinical outcomes and reduce mortality. | 5 | 5 | Goyal et al., 2015 |
|  | In selected patients with AIS from anterior circulation proximal LVO of the ICA or M1, presenting between 6 and 24 hours from onset of symptoms, with age <80 years, NIHSS score ≥6, prestroke mRS score 0 to 1, ASPECTS 3 to 5, and without significant mass effect on imaging, EVT is recommended to improve functional clinical outcomes and reduce mortality. | 5 | 5 | Jovin et al., 2015 |
|  | In selected patients with AIS from anterior circulation proximal LVO of the ICA or M1 presenting within 6 hours from onset of symptoms, with age <80 years, NIHSS score ≥6, prestroke mRS 0 to 1, ASPECTS 0 to 2, and without significant mass effect on imaging, EVT is reasonable to improve functional clinical outcomes and reduce mortality. | 4 | 4 | Saver et al., 2015; Campbell et al., 2015; Bracard et al., 2016; Berkhemer et al., 2015; Sarraj et al., 2023; Huo et al., 2023; Costalat et al., 2024; Yoshimura et al., 2022; Bendszus et al., 2023 |
| **2026** | In patients with AIS from anterior circulation proximal LVO of the ICA or M1 presenting between 6 and 24 hours from onset of symptoms, with NIHSS score ≥6, prestroke mRS score 0 to 1 and ASPECTS ≥6, EVT is recommended to improve functional clinical outcomes and reduce mortality. | 4 | 4 | Nogueira et al., 2018; Albers et al., 2018 |
|  | In patients with AIS from anterior circulation proximal LVO of the ICA or M1 presenting within 6 hours from onset of symptoms, with NIHSS score ≥6, and ASPECTS of ≥6, who have a prestroke mRS score of 3 to 4, EVT might be reasonable to improve functional clinical outcomes and reduce accumulated disability. | 4 | 4 | Benali et al., 2023 |
|  | In patients with AIS from occlusion of the dominant proximal M2 division of the MCA presenting within 6 hours from onset of symptoms with a prestroke mRS score of 0 to 1, NIHSS score of ≥6, and ASPECTS of ≥6, EVT is reasonable to improve functional outcomes, but the benefits are uncertain. | 4 | 4 | Goyal et al., 2025 |
|  | In patients with AIS from occlusion of the proximal nondominant or codominant division proximal M2 segment of the MCA, or distal MCA, anterior cerebral artery (ACA), or posterior cerebral artery (PCA), EVT is not recommended to improve functional outcomes. | 3 | 3 | Psychogios et al., 2025 |
|  | In patients with AIS, with basilar artery occlusion, a baseline mRS score of 0 to 1, NIHSS score ≥10 at presentation, and PC-ASPECTS ≥6 (mild ischemic damage), EVT within 24 hours from onset of symptoms is recommended to achieve better functional outcome and reduce mortality. | 5 | 5 | Jovin et al., 2022; Tao et al., 2022 |
|  | In patients with AIS, with basilar artery occlusion, a baseline mRS score of 0 to 1, NIHSS score 6 to 9 at presentation, and PC-ASPECTS ≥6 (mild ischemic damage) the effectiveness of EVT within 24 hours to improve functional outcomes and reduce mortality is not well established. | 3 | 3 | Renu et al., 2022; Langezaal et al., 2021 |
|  | In patients with AIS from occlusion of medium or distal vessels of the anterior, middle (nondominant or codominant M2, M3), or posterior cerebral arteries, EVT with stent retrievers is of no benefit for improving functional outcomes. | 5 | 5 | Psychogios et al., 2025; Goyal et al., 2025 |
|  | In patients with AIS who achieve complete or near-complete EVT (modified TICI 2c or greater), the administration of adjunctive intraarterial thrombolytics with urokinase, alteplase, or tenecteplase may be reasonable to improve cerebral reperfusion and 90-day functional outcome. | 4 | 3 | Renu et al., 2022; Liu et al., 2025; Hu et al., 2025; Huang et al., 2025 |
|  | In the management of patients with AIS in the setting of LVO, preoperative administration of tirofiban before EVT is not useful to improve 90-day functional outcome. | 2 | 2 | Qiu et al., 2022 |
|  | In patients with recent (<24 hours) minor (NIHSS score ≤5) noncardioembolic AIS or high-risk TIA (ABCD2 score ≥6 or symptomatic intracranial or extracranial ≥50% stenosis of an artery that could account for TIA) who did not receive IVT, DAPT with ticagrelor (including loading dose) plus aspirin for 30 days may be considered to reduce the risk of 30-day recurrent stroke. | 4 | 4 | Johnston et al., 2020 |
|  | In patients with minor (NIHSS score ≤5) noncardioembolic AIS or high-risk TIA (ABCD2 score >4) within 24 to 72 hours from stroke onset, or NIHSS score of 4 to 5 within 24 hours from onset, who did not receive IVT, with presumed atherosclerotic cause (>50% stenosis of intracranial or extracranial stenosis that was likely to have accounted for clinical presentation or acute new infarctions on imaging of presumed large artery atherosclerosis origin), DAPT (clopidogrel and aspirin) for 21 days followed by SAPT is reasonable to reduce the 90-day risk of recurrent stroke. | 4 | 4 | Gao et al., 2023 |
| **2026** | In patients with minor (NIHSS score ≤3) noncardioembolic AIS or high-risk TIA (ABCD2 score >4) within 24 hours after symptom onset who did not receive IVT and who carry the CYP2C19 loss-of-function allele, DAPT with ticagrelor and aspirin for 21 days (followed by ticagrelor monotherapy) may be reasonable in preference over DAPT with clopidogrel and aspirin to reduce the 90-day risk of recurrent stroke. | 4 | 4 | Wang et al., 2021 |
|  | In carefully selected (eg, milder severity) patients with AIS with atrial fibrillation, a strategy of early oral anticoagulation poststroke is low risk and is reasonable compared with a strategy of delayed anticoagulation, although the efficacy of early anticoagulation for prevention of early recurrent stroke is not established. | 4 | 4 | Fischer et al., 2023; Werring et al., 2024; Oldgren et al., 2022 |
|  | In patients with AIS who experience HT, initiation or continuation of anticoagulation may be considered depending on the specific clinical scenario and underlying indication. | 3 | 3 | Gioia et al., 2016; England et al., 2010 |
|  | In patients with AIS, mechanical hemodynamic augmentation with counterpulsation devices or sphenopalatine ganglion stimulation is not recommended to improve functional clinical outcomes. | 1 | 1 | Guluma et al., 2015; Bornstein et al., 2019 |
|  | In patients with stroke with dysphagia, treatment with pharyngeal electrical stimulation (PES), can be beneficial to reduce dysphagia severity and decrease the risk of aspiration. | 3 | 3 | Jayasekeran et al., 2010 |
|  | In patients with severe stroke with dysphagia requiring tracheotomy and mechanical ventilation, treatment with PES, after ventilator weaning can be beneficial to decrease dysphagia severity, reduce the risk of aspiration, and expedite decannulation. | 3 | 3 | Suntrup et al., 2015; Dziewas et al., 2018 |
|  | In patients with large hemispheric infarction aged 18 to 70 years, the use of IV glibenclamide does not result in improved functional outcome and is not recommended. | 1 | 1 | Sheth et al., 2024 |

RCT = Randomized controlled trial; AIS = Acute Ischemic Stroke; AHA = American Heart Association; ASA = American Stroke Association. New recommendations from 2018 and 2026 AHA/ASA AIS guidelines; clinical impact scores (1=lowest to 5=highest); RCT references reflect primary supporting trials.

**Supplemental Table S2. Distribution of Randomized Clinical Trials by Research Area and Class of Recommendation for New Recommendations in 2018 and 2026 AHA/AIS AIS Guidelines**

| **Section** | **COR** | **2018** | **2026** |
| --- | --- | --- | --- |
| **Endovascular Therapy** | I | 0 | 6 |
|  | IIA | 5 | 10 |
|  | IIB | 3 | 7 |
|  | III | 0 | 4 |
|  | **Total** | **8** | **27** |
| **Imaging Use** | I | 2 | 0 |
|  | IIA | 0 | 4 |
|  | IIB | 0 | 2 |
|  | III | 7 | 0 |
|  | **Total** | **9** | **6** |
| **Poststroke Care** | I | 0 | 0 |
|  | IIA | 5 | 7 |
|  | IIB | 12 | 4 |
|  | III | 1 | 3 |
|  | **Total** | **18** | **14** |
| **Prehospital/System Care** | I | 4 | 4 |
|  | IIA | 0 | 1 |
|  | IIB | 0 | 4 |
|  | III | 0 | 8 |
|  | **Total** | **4** | **17** |
| **Thrombolysis** | I | 0 | 5 |
|  | IIA | 0 | 2 |
|  | IIB | 7 | 7 |
|  | III | 1 | 11 |
|  | **Total** | **8** | **25** |
| **Total** |  | **47** | **89** |

COR = Class of Recommendation (I=Strong; IIA=Moderate; IIB=Weak; III=No benefit/Harm).

Supplemental Figure S1. Distribution of randomized controlled trial (RCT) funding sources cited in the 2018 and 2026 American Heart Association/American Stroke Association (AHA/ASA) acute ischemic stroke (AIS) guidelines. Stacked bar graphs show the distribution of RCTs cited in the 2018 and 2026 guidelines across three analytic approaches: unweighted analysis, reference-density-weighted analysis, and clinical-impact-weighted analysis. Within each analytic approach, bars represent the percentage contribution of trials by funding source: government/academic/nonprofit vs industry (private/mixed). Differences between guideline eras were not statistically significant across analyses (unweighted: p=0.471; reference-density-weighted: p=0.557; clinical-impact-weighted: p=0.644). Percentages were calculated using the number of RCT references in each funding category (numerator) divided by the total number of RCT references supporting new recommendations for that year and analytic approach (denominator): unweighted (2018: N = 47; 2026: N = 89), reference-density-weighted (2018: N = 21; 2026: N = 45), and clinical-impact-weighted (2018: N = 65; 2026: N = 148.5).
